# Exposure of Live-Line Workers to Magnetic Fields: A Dosimetric Analysis

**DOI:** 10.3390/ijerph17072429

**Published:** 2020-04-02

**Authors:** Oriano Bottauscio, Alessandro Arduino, Davide Bavastro, Davide Capra, Arianna Guarneri, Alessandro A. Parizia, Luca Zilberti

**Affiliations:** 1 Istituto Nazionale di Ricerca Metrologica, 10135 Torino, Italy; a.arduino@inrim.it (A.A.); l.zilberti@inrim.it (L.Z.); 2 Terna Rete Italia S.p.A., 00156 Roma, Italy; davide.bavastro@terna.it (D.B.); alessandro.parizia@terna.it (A.A.P.); 3 CESI S.p.A., 20134 Milan, Italy; davide.capra@cesi.it; 4 Terna S.p.A., 00156 Roma, Italy; arianna.guarneri@terna.it

**Keywords:** live-line working, electromagnetic fields, human exposure, dosimetry analysis

## Abstract

In this paper the authors present the results of a dosimetric analysis related to the exposure of live-line workers to the magnetic fields generated by high voltage overhead lines and substations. The study extends the work published by Dawson et al. in 2002, considering more evolved anatomical models nowadays available, the new reference limits given by the 2013/35/EU Directive, and a new methodology, based on the intercomparison of two alternative solvers and the use of data filtering. Moreover, additional exposure scenarios are here considered with respect to the studies already available in literature. The results show that for the exposure scenario of high voltage live line works with bare hand method, in any analyzed position, the exposure limits for the tissues of the central nervous system, as well as for all other tissues, are never exceeded, despite in some cases the action levels are exceeded. For the exposure of workers in substations near 220 kV and 380 kV line trap coils exposure is compliant with the regulatory limits if the current flowing through the line trap does not exceed the value of 1000 A. Finally, for the exposure of workers in substations near cable connections, electric field values induced in the body are always lower than regulatory limits with a phase current value equal to 1600 A r.m.s.

## 1. Introduction

Live-line working is a technique of maintenance on high voltage (HV) overhead lines kept in service; it allows increasing electrical plant reliability by reducing or eliminating line outages. Although it is more hazardous for personnel than working with the electrical equipment switched-off, this approach is used in the electrical transmission and distribution systems to avoid the economic costs of turning off power to customers in order to perform essential periodic maintenance on transmission lines and equipment.

At present two techniques of live-line works are applied worldwide: the “distance technique” (also called “hot sticks”) and the “bare hand method” (or “contact technique”). The procedures associated to the first technique requires that workers are directly kept at the ground potential and operate on live parts by using special insulating tools. The “contact technique”, which is typically used for 220 kV and 380 kV lines, requires that workers, who wear special conductive clothing, reach the same potential as the live parts on which they must operate using hand-maneuvered metal tools.

During all live-line working, the operators work in close proximity to live conductors, and therefore are exposed to high electromagnetic fields (EMFs). Even if during “contact techniques” workers wear special conductive clothing, that protects them against the exposure to the electric field (see for example [1]), they are in any case exposed to magnetic fields that consequently induce currents in the worker’s body.

In 2013, the European Directive 2013/35/EU [2] laid down minimum requirements for the protection of workers from risk to their health and safety arising, or likely to arise, from exposure to electromagnetic fields, with reference both to biophysical direct effects and to indirect effects. In particular, the Directive establishes exposure limit values (ELVs) to the electric field induced within the body with the aim of avoiding acute health and sensory effects in the human body. Sensory ELVs apply to tissues of central nervous system (CNS) as they refer to sensory perceptions and minor changes in brain function; health ELVs apply to all tissues of head and body as they refer to thermal heating (at higher frequencies) or stimulation of nerve and muscle tissues (at lower frequencies).

The Directive also sets ‘action levels’ (ALs) for time varying magnetic fields, formulated to simplify the process of demonstrating compliance with the relevant ELVs, allowing a direct exposure assessment via measurement of the magnetic field in the considered environment. ALs, where appropriate, indicate a threshold above which employers must take one or more of the protection or prevention measures specified in the Directive. The rationale of the Directive is represented by the recommendations of the International Commission on Non-Ionizing Radiation Protection (ICNIRP) for time varying electric and magnetic fields (1 Hz to 100 kHz) [3] and for static fields [4]. These documents introduced basic restrictions (BRs) for induced field within the human body and reference levels (RLs) for external applied fields.

Following the Directive specifications, compliance with the ALs provided by the Directive will ensure compliance with the relevant ELVs in all non-uniform exposure conditions; whenever the ALs are exceeded, compliance with the relevant ELVs needs to be demonstrated. Anyway, in the case of a very localized source within a distance of a few centimetres from the body, the induced electric field shall be determined dosimetrically, case by case and this is the exposure scenario for live-line working.

The exposure to electromagnetic fields produced by high-voltage facilities was considered in several published papers [5,6,7,8,9,10,11,12], focusing the attention to the electric and magnetic field strength, either by on-site measurements or by simulations. Different transmission lines configurations or exposure scenarios in substation were analyzed. Occupational exposure assessment is also considered in many other studies (see for example [13,14]).

In order to ensure that ELVs are not exceeded during operation due to magnetic field, the grid operators impose limits to the current in the conductors while live work is in progress. Detailed dosimetric analysis is needed to define, for each exposure scenario, the relationship between the magnitudes of the current flowing in the conductors (and, hence, of the magnetic field) and of the electric field induced within the body, to establish maximum values of current which can circulate in conductors to guarantee the respect of ELVs in the workers body. Although also for “distance techniques” the electric field is of relevance and requires assessment, the present study focuses on magnetic field exposure in occasion of live-line working “contact techniques” and for works close to energized HV cables.

A relevant study of the magnetic field exposure for live-line working was published by Dawson et al., in 2002 [15]. In this work, dosimetry is evaluated for live-line workers exposed to 50 Hz non-uniform magnetic fields from typical high-voltage transmission lines in the United Kingdom, considering different overhead line bundles (twin-, triple- and quadruple-conductor transmission line). The analysis was developed adopting a human model (having 80 different tissues) derived from two of the most detailed anatomical models available at that time, the one obtained from Yale Medical School (Zubal et al. [16]) and the Visible Human data set of the US National Library of Medicine [17]). The dosimetric analysis was performed with reference to the 1998 edition of the ICNIRP Guidelines [18], where basic restrictions were given in terms of induced current density within the body, but the Authors analyzed also the levels of induced electric field, anticipating that the electric field was a more suitable indicator than the current density, as adopted in the successive edition of the ICNIRP Guidelines. The paper of Dawson also considered the issues related to staircase errors at tissue interfaces and averaging methods, concluding that the 99th percentile was the most reliable metric for dosimetric purposes.

Other dosimetric studies have been published subsequently, focusing on specific transmission tower configurations (see for example [19,20,21,22]) or using simplified models of the human body [23,24,25], as indicated by the IEC/EN 62233 Standards [26]. Examples of dosimetric studies, but restricted to the exposure to high electric fields, can be found in [27,28].

During the last decade, the availability of reliable anatomical human models is progressively increased, making these models becoming more and more anatomically realistic and representative of entire ‘human families’. It is worth mentioning here the NORMAN (it stands for NORmalized MAN (it stands for aNAtOMIcal model) and NAOMI datasets [29,30], the TARO and HANAKO models (developed by the National Institute of Information and Communications Technology) [31], representative of Japanese men and women, the Virtual Family models [32], nowadays largely adopted in different field of applications, the 4D Extended Cardiac-Torso (XCAT) Phantoms [33] and the Maxwel model [34], which are the only models based on a realistic representation of the surfaces of the different organs, whereas all the other models previously cited are voxelised (i.e., made of elementary cubes) and intended to be used preferentially by calculation methods based on hexahedral meshes. An extensive review of the effect of the use of different anatomical models has been published by Magne and Deschamps [35]. They have analyzed published dosimetric studies of anatomically realistic models of human bodies exposed to 50/60 Hz uniform fields and analyzed the differences in terms of uncertainty components of dosimetric calculations.

In parallel to the evolution of the anatomical models, numerical tools for computational dosimetry have had an extraordinary development, by virtue of the potentiality of last generation processors, the software development and the analysis of the accuracy of the dosimetric results (e.g., [36,37,38,39,40]). The latter includes the detection of possible numerical artefacts, which is also a topic of great interest, particularly when a comparison with exposure limits is needed [41,42,43].

In this work, an analysis of exposure of workers to magnetic fields during live-line working “contact techniques” on HV overhead lines at power frequency (50 Hz) is presented, based on an approach similar to the analysis done by Dawson et al. in 2002 [15], but with the following novelties:

▪ More evolved anatomical models adopted, making reference to the version 3.1 of the Virtual Population [32];
▪ The 2013/35/EU Directive, referring to health and sensory ELVs, taking also into account suggestions provided by the 2010 ICNIRP Guidelines;
▪ More evolved filtering techniques applied to filter the raw computed data of the induced electric field, before comparing them with the ELVs;
▪ New scenarios of workers exposure.

The same analysis is also extended to the exposure of workers to magnetic fields during works close to energized HV cables. 

The paper is organized as follows: Section 2 describes all methodological aspects adopted for the analysis. In particular, the adopted anatomical models are introduced in Section 2.1, while the numerical methods for the dosimetric analysis (i.e., the computation of the induced electric field within the human body) are briefly described in Section 2.2. The metric adopted to analyze the exposure is discussed in Section 2.3, where the need of filtering the numerical raw data is discussed. Finally, the considered exposure scenarios are detailed in Section 2.4.

Section 3 focuses on the analysis and discussion of the results of the dosimetric simulations. First part is focalized to the estimation of the uncertainty associated with the numerical results, with the aim of providing a safety factor for the comparison with exposure limits. Second part shows the analysis of each single exposure scenario and posture, comparing the outcomes of the simulations with the health and sensory ELVs.

## 2. Materials and Methods

The analysis is performed solving the electromagnetic field problem within the human body model exposed to the magnetic field generated by the considered source (overhead transmission line, line trap coils, substation connections) of each exposure scenario, as detailed in Section 2.4. For each field source, a realistic virtual model of the conductors has been generated, discretizing the conductors in 8-nodes and 20-nodes hexahedra (the latter have curved edges), suitable for computing the magnetic flux density by Biot-Savart law. These B-field values are used as input for the electromagnetic solver used for the in-silico simulations. In the next subsections details of the modelling approach are given, together with a description of the considered exposure scenarios.

### 2.1. Model for in-Silico Simulations

The analysis has been performed using the ‘Duke’ model belonging to the Virtual Population (ViP 3.1) developed by the IT’IS Foundation [32]. This model, here used to simulate a worker, refers to a 34-years old adult male, with height 1.77 m and weight 70.3 kg, composed of 318 different anatomical parts grouped in 74 biological tissues. 

Among all identified tissues, the following ones are assumed to belong to the central nervous system (CNS), when comparing the results with the sensory effects ELVs: Brain (White and Grey Matter), Cerebellum, Cerebrospinal Fluid, Hippocampus, Midbrain, Commissura Posterior, Commissura Anterior, Spinal Cord. Health ELVs apply to all other tissues.

The static version of this anatomical model has been used without modifying its posture, which is acceptable for the considered exposure scenarios. The electrical properties of the tissues have been derived from the IT’IS Database [44], assuming the set of values specific for low frequency dosimetry. The electrical conductivity values of most significant tissues are reported in Table 1; they range from 0.0035 S/m (bones) to 1.777 S/m (cerebrospinal fluid). The skin conductivity is set to 0.17 S/m to take into account the presence of the deep granular tissue, i.e., the dermis [29,45]. The model was discretized with a uniform voxel mesh at a resolution of 2 mm, giving rise to a total number of voxels equal to about 8.41 × 10^6^.

In the present study, the electrical properties of tissues have been assumed to be isotropic. This assumption is commonly adopted in numerical dosimetry, even if, especially at low frequency, some tissues exhibit anisotropic properties. This effect is particularly relevant for tissues as bone and skeletal muscle. As an example, for skeletal muscle the conductivity can be up to 10 times lower along the length of the muscle fibres compared to the perpendicular orientation [46]. Very rare data can be found in literature about the impact of this simplifying assumption on dosimetric results. In [47], it is reported that heterogeneous anisotropic tissues can have an impact on the distribution of the electric field up to ~20%. In the absence of reliable measured data on the electrical anisotropy of all considered tissues, this effect has been disregarded in the present study, keeping in mind that results in some tissues could be affected by an additional error of 20% at the maximum.

### 2.2. Finite Element Solvers for the Dosimetric Analysis

The in-silico dosimetric simulations have been performed using two solvers based on complementary field formulations [40] in order to test their accuracy and provide a sort of range of variability of the results. The first solver adopts an edge-based finite element method, assuming an electric vector potential ***T*** (***J*** = curl***T***) as the unknown (***T***-formulation). Alternatively, a nodal finite element solver, based on the ***A***-*ϕ* formulation, was used assuming the electric scalar potential *ϕ* (***E*** = grad*ϕ−jω**A***_s_) as unknown.

The weak form equations for the domain Ω (i.e., the human body model) are, respectively:

(1) $$\int_{\Omega }\frac{1}{\sigma }\mathrm{curl}Τ\cdot \mathrm{curl}\upsilon \;dv=-j\omega \mu_{0}\int_{\Omega }H_{s}\cdot \upsilon dv$$

and:

(2) $$\int_{\Omega }\sigma \mathrm{grad}\phi \cdot \mathrm{grad}w\;dv=j\omega \int_{\Omega }\sigma A_{s}\cdot \mathrm{grad}w\;dv$$

being *σ* the piecewise constant electrical conductivity of tissues, *ω* the angular frequency of the driving terms (magnetic field ***H****_s_* or magnetic vector potential ***A****_s_*), *μ*_0_ the magnetic permeability, *j* the imaginary unit. In (1) and (2) ***υ*** and *w* are the vector and scalar test functions, respectively. Equations (1) and (2) are verified for any test function ***υ*** and *w*. Induced currents are assumed to be confined within the body. This results to be implicit in (1), thanks to the Dirichlet boundary condition ***n*** × ***T*** = 0; likewise, in (2) this is guaranteed by the Neumann boundary condition grad*ϕ*·***n*** = *jω**A**_s_*·***n***, being ***n*** the outward normal unit vector to the boundary.

Problems (1) and (2) are numerically solved by using a finite element discretization into hexahedra coincident with the human body voxels. Due to the large number of unknowns, the algebraic system of equations is solved through a generalized minimal residual method (GMRES) algorithm [48], which allows avoiding the storage of the problem matrix. Electric field magnitudes induced in the barycenter of each voxel of the anatomical model are computed as:

(3) $$E=\frac{1}{\sigma }\mathrm{curl}T,\;\mathrm{for}\;\mathrm{the}\;T\text{-}\mathrm{formulation}$$

(4) $$E=\mathrm{grad}\phi -j\omega A_{s},\;\mathrm{for}\;\mathrm{the}\;A\text{-}\phi \;\mathrm{formulation}$$

Both formulations are implemented using an ungauged approach, considering all internal edges or all nodes as unknowns. This choice increases the number of unknowns but favors the convergence of the iterative solver [49]. 

### 2.3. Numerical Artefacts and Filtering Techniques

It is well documented that numerical solutions computed on voxel-based models can be affected by numerical artefacts (see for example [37,39,41]). Main causes are the stair-casing error [39], unavoidable when using voxelized models, and the contrast of electromagnetic properties of adjacent voxels (at the tissue boundaries).

An example of quantification of the stair-casing error introduced in voxelized domains is reported in [42]. In addition, local artefacts may occur in proximity of the skin, where the voxelization process may introduce local contacts not present in the original Computer-aided design (CAD) model, e.g., between the legs (with reference to skin-to-skin contact voxels, see the approved Draft of the IEEE C95.1 Standard [43]). 

All these effects introduce fictitious outliers in the solution (i.e., the induced E-field values in the human body), which have to be avoided, particularly when they alter the maximum level of the induced electric field that is the metric to be used for evaluating the compliance with the ELVs given in the Directive. The Directive itself takes this aspect into consideration. When compliance with the ELVs has to be verified, the assessment shall take into account uncertainties of measurements or calculations, such as numerical errors, source modelling, phantom geometry and electrical properties of tissues and materials, determined in accordance with relevant good practice.

Suggestions for overcoming numerical errors in dosimetric results are given in the ICNIRP Guidelines. The document proposes two strategies: (a) averaging the computed electric field on a 2 × 2 × 2 mm^3^ voxel and (b) adopting the 99th percentile of the induced electric field magnitude, instead of its absolute maximum as determined by the simulations. While the averaging over a 2 × 2 × 2 mm^3^ volume contributes to smoothing local unphysical outliers, the adoption of the 99th percentile is more questionable. In fact, previous studies (e.g., [45,50,51]) have put in evidence some faults of such an approach, which may overestimate the presence of outliers in the case of a strongly heterogeneous exposure.

In all these cases, the application of the 99th percentile (for the whole body, or for a very extensive specific tissue, like the skin) may introduce an overcorrection and remove genuine “hot-spots”, which should be taken into account in the exposure assessment. For instance, the hand of a person handling an electrical device that produces a magnetic field results to be much more exposed to the field itself than the rest of the body. In this case, the application of the 99th percentile may introduce an overcorrection mistakenly removing real “hot-spots”.

To overcome some of the flaws in the metric recommended by ICNIRP, another way for removing outliers, more conservative with respect to the 99th percentile, has been proposed in [41] and extended in [42]. In the present study, we adopt such a strategy and we compare the results with those coming from the use of the 99th percentile.

For each simulation, the dataset of the magnitude of all ***E***-field values (one for each voxel) is collected and the values are sorted in ascending order, despite their spatial distribution. The obtained curve typically exhibits a strong upward concavity due to the sudden rise of the highest values. The “gradient” of the curve (defined as the difference between two adjacent values divided by their mean) is computed for the last percentile of the sorted data. The frequency distribution of the logarithm of the gradient is then plotted. In many cases the latter distribution shows an almost symmetric and unimodal shape. By considering an interval corresponding to three times the standard deviation (for a Gaussian distribution, this choice covers 99.87% of the smallest gradient values), the detection point (DP) of the outliers is determined and we search, within the last percentile of the sorted ***E***-field values, the first pair of values that produce a gradient whose logarithm is larger than DP. From these values on, the computed ***E***-field values are considered as outliers and are corrected.

The correction is obtained by interpolating the curve on the last non-outliers, whose number is chosen equal to the number of the outliers to be corrected and extrapolating this trend. This choice, which constitutes the main improvement with respect to [41], guarantees a good continuity of the resulting curve, as well as of its first derivative, at the interface between the points kept at their original values and those obtained via extrapolation.

### 2.4. Exposure Scenarios

Three exposure scenarios are considered in this study: (A) exposure of workers near HV overhead lines, (B) exposure of workers in substations near line trap coils and (C) exposure of workers in substations near cable connections. The positions (A) and (B) are the heavy conditions where the HV live operators work with bare hand method and they are nearly close to the source. The position (C) is a particular situation where operators make a check on the HV cables; in particular is analyzed the worst possible exposure that occurs near the HV cable bushing, where the three conductors are widely spaced to ensure the air insulation condition. 

**Figure 1:**
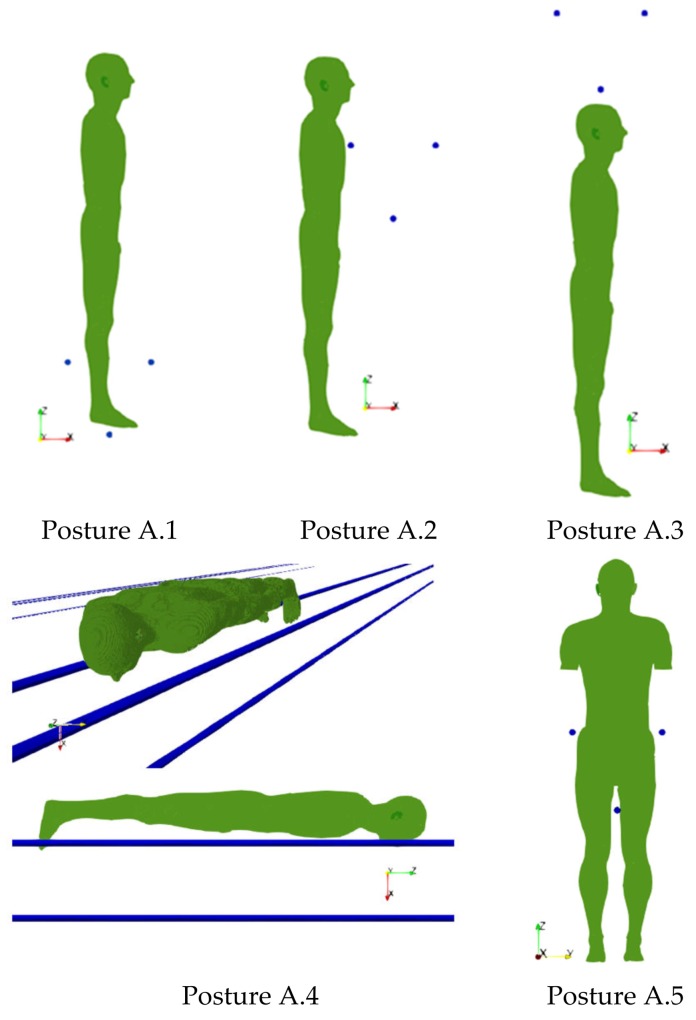
Five considered positions of the human body with reference to the exposure scenario A. The traces of the conductors are also reported in the figures.

For the first scenario, a 380 kV overhead line with triple bundles per phase is considered. The conductor diameter is 31.5 mm and the triple sub-conductor centers lie at the vertices of an inverted equilateral triangle with 400 mm edges. The three phases are almost aligned at a distance of 9.54 m. The rated phase current is assumed to be 2950 A r.m.s. in accordance with the CIGRE (Conseil International des Grands Réseaux Électriques) Guidelines [52]. This value represents the maximum nominal current flowing through a bonding conductor of an HV overhead line 380 kV in Italy. Five different positions of the human body are considered (from A.1 to A.5), mimicking realistic positions during live-line working. They are shown in Figure 1.

In posture A.1, the worker is standing over a bundle, with the feet at 15 mm from the lower conductor. In posture A.2 the worker is facing the bundle with a distance between conductor and thorax of 10 mm. For posture A.3 the bundle is over the worker’s head at a minimum distance of 50 mm. The worker lies down the bundle, aligned with the conductors, in posture A.4; the minimum distance between conductor and thorax is 10 mm. Finally, posture A.5 simulates the worker sitting on the trolley over the bundle. To avoid the use of postured anatomical models, the body is located as depicted in Figure 1. The distance between the lower conductor and the body is 100 mm, while the lateral distance between body and conductors is 10 mm. The arms have been intentionally removed to avoid unphysical intersections with the lateral conductors; this simplification will not alter the relevance of the results considering that the highest induced electric field values are not reached in the arms.

**Figure 2:**
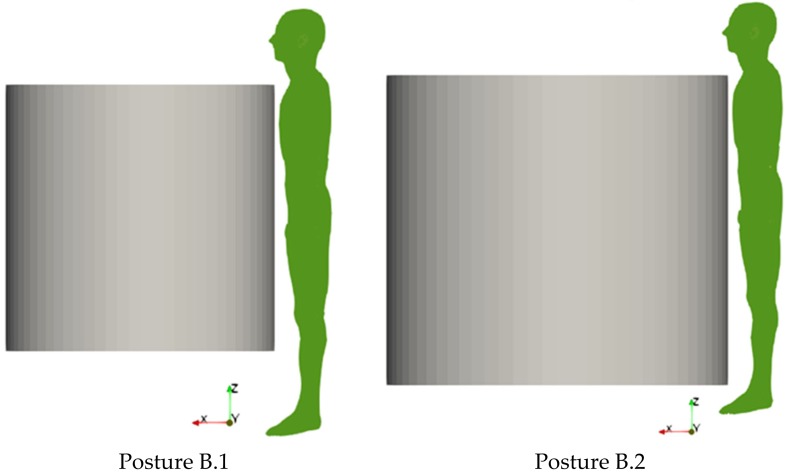
Two considered positions of the human body with reference to the exposure scenario B. On the left the case of 220 kV line trap coil (B.1), on the right the case of the 380 kV line trap coil (B.2).

For the second exposure scenario, the worker is placed in proximity of a line trap coil. Two situations have been analyzed: (B.1) a 220 kV line trap coil and (B.2) a 380 kV line trap coil. The rated phase currents are 2000 A r.m.s. and 3150 A r.m.s., respectively for the 220 kV coil and the 380 kV coil. The coil geometrical data are summarized in Table 2. In both postures B.1 and B.2 the worker is standing with a distance between his thorax and the coil of 10 mm (see Figure 2).

**Figure 3:**
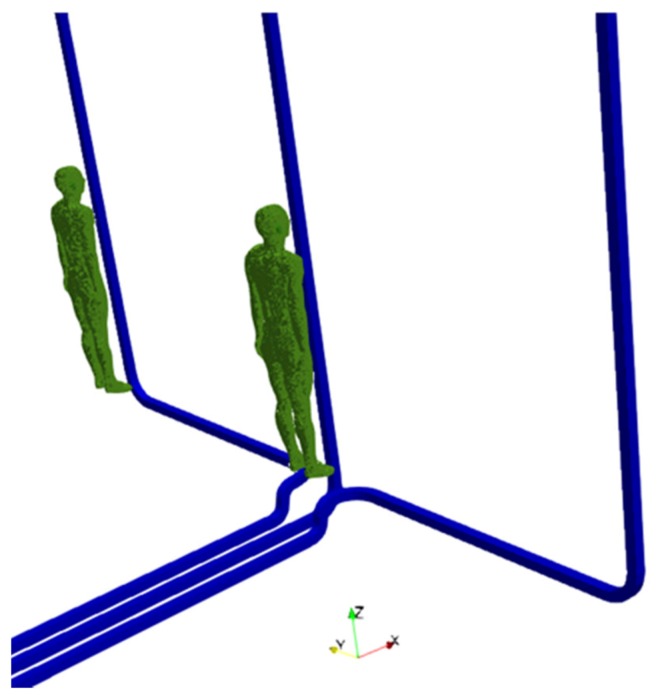
Two considered positions of the human body with reference to the exposure scenario C.1, with the human body close to the central (C.1a) or lateral (C.1b) phase conductor. The traces of the conductors are also reported in the figure.

For the third exposure scenario, the worker is placed near cable connections belonging to a substation. Two configurations have been analyzed: (C.1) phase conductors at a distance of 2.5 m and (C.2) phase conductors at a distance of 5 m. The rated phase current is equal to 1600 A r.m.s. For both configurations, two postures of the worker are considered: worker standing in front of the central conductor (C.1a and C.2a) or in front of a lateral conductor (C.1b and C.2b). The distance between thorax and conductor is 70 mm. The postures C.1a and C.1b are shown in Figure 3.

For each exposure scenario, the maximum values of E-field have been calculated, filtering the value using the 99th percentile (E_99) and using the algorithm described in Section 2.3) (E_out). These values are compared with the sensory effects ELVs (0.14 V/m) of the Directive for the CNS tissues and with the health effects ELVs (1.1 V/m) for all other tissues. In particular, we refer to the limits expressed in terms of peak values instead of r.m.s. values, that is more significant in case of elliptical polarized fields due to three phase sources.

## 3. Results and Discussion

### 3.1. Considerations about Results Accuracy

For each considered exposure scenario and posture, the values of the E-field induced within the body have been computed applying the two complementary solvers described in Section 2.2. Taking advantage of the linearity of the problem, simulations have been run assuming a unitary current (peak value of the sinusoidal waveform) in each conductor, with the proper phase shift. Results can be successively rescaled to the desired current amplitude (e.g., rated phase currents).

**Figure 4:**
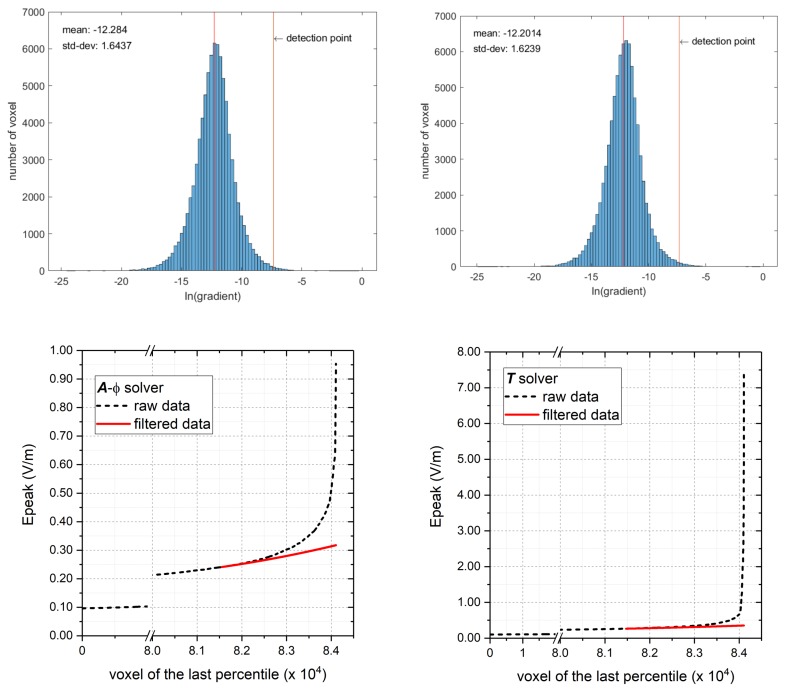
Example of data filtering applied to raw data (values obtained with a unitary current amplitude imposed to the conductors) computed with the two solvers for Posture A.2. On the upper plots, the frequency distributions of the natural logarithm of the gradient of the sorted values of the E-field magnitude. On the lower plots the correction of data applying the filtering technique. On the left the data for the ***A***-*ϕ* solver, on the right the data for the ***T*** solver.

The raw values obtained as outcomes of the two solvers (E_raw_T_ and E_raw_A-_*_ϕ_*) are elaborated using the filtering technique for outlier removal described in Section 2.3, obtaining the datasets (E_out_T_ and E_out_A-_*_ϕ_*). An example of the filtering procedure is depicted in Figure 4, which refers to the exposure scenario A, with posture A.2. 

The figure shows the frequency distributions of the natural logarithm of the gradient of the sorted values of the E-field magnitude (raw data), restricted to the last percentile. The distributions are almost identical for the ***A***-*ϕ* and ***T*** solvers, evidencing the overall general agreement between the two formulations. The corresponding detection point (DP) is evidenced, allowing the determination of the outliers that must be corrected by the algorithm. In the same figure, the last percentile of E-field values (sorted in the ascending order) are plotted for the two datasets, showing that the T solver leads to higher values of the outliers (the maximum is 7.36 V/m, against 0.95 V/m for the other dataset). This large discrepancy reduces after applying the filtering technique. In fact, after outlier removal and extrapolation the maximum values obtained with the two solvers become much more coherent (0.318 V/m for the ***A***-*ϕ* solver and 0.354 V/m for the ***T*** solver), with discrepancies of about 10%. Similar behaviour is found for all simulations considered in this study. A summary of the maximum values of the induced E-field after filtering is reported in Table 3. It results from the analysis of the data collected that higher values can be provided by one solver or the other, so that the ratio between maximum E-field obtained with the ***T*** solver (E_out_T_) and the one obtained with the ***A***-*ϕ* solver (E_out_A-_*_ϕ_*) ranges between a minimum of 0.72 to a maximum of 1.62. In the same Table, for each case, the 99th percentile of the E-field values are reported (E_99_T_ and E_99_A-_*_ϕ_*) in accordance with the suggestions of ICNIRP. These values are considerably lower with respect to E_out values and very close for the two solvers. The range of variability of the ratio between the values obtained by the two solvers reduces and ranges from 0.93 to 1.08. Despite the lower variability of the data, these results cannot be considered more reliable because of a possible over-filtering effect.

**Figure 5:**
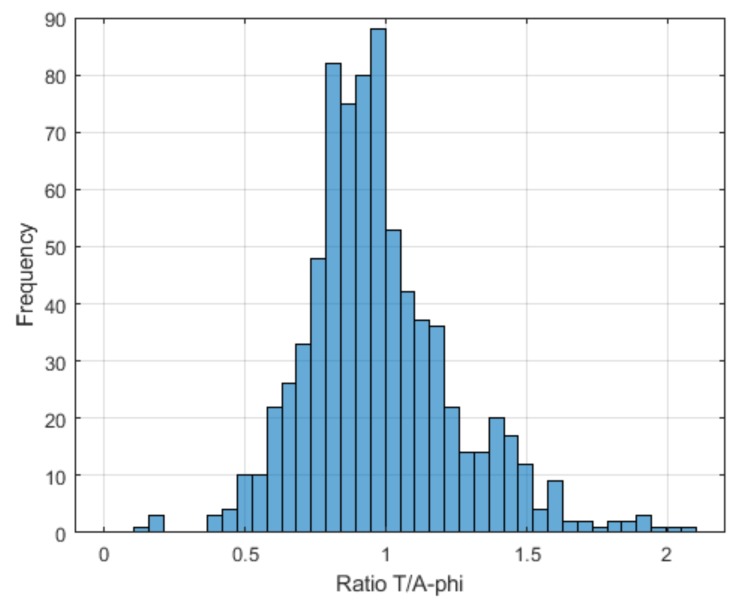
Frequency distribution of entire set of ratios between maximum E-field values (after filtering) obtained with ***T*** solver (E_out_T_) with respect to the ***A***-*ϕ* solver (E_out_A-_*_ϕ_*), computed for each tissue.

In order to give an estimate of the uncertainty associated to the maximum E-field values (after filtering) provided by the two solvers, the frequency distribution of the ratios between maximum E-field values obtained by the two solvers within each single tissue (see Figure 5) has been analyzed. The average value of the distribution is 1.07, showing that an overall agreement between the two solvers is generally ensured. The probabilistically symmetric 95% coverage interval is between a ratio of 0.52 and 2.16.

The above interval provides an estimate of the reliability of the numerical results useful when comparing maximum computed values with reference limit values provided by the standards. This means that, if the analysis id developed by using just one of the two solvers, a safety factor of ~2 has to be applied to include all possible unpredictable errors introduced by the numerical model.

By adopting this concept, in the next sessions the analysis of the different exposure scenario has been developed by referring to the ***A***-*ϕ* solver only and the results reported should be interpreted as affected by an uncertainty correlated to the coverage interval identified above. It must be remarked that these considerations cannot be extrapolated to any type of dosimetric analysis but keep their validity only for similar exposure scenarios.

### 3.2. Results about the Exposure Scenario A

**Figure 6:**
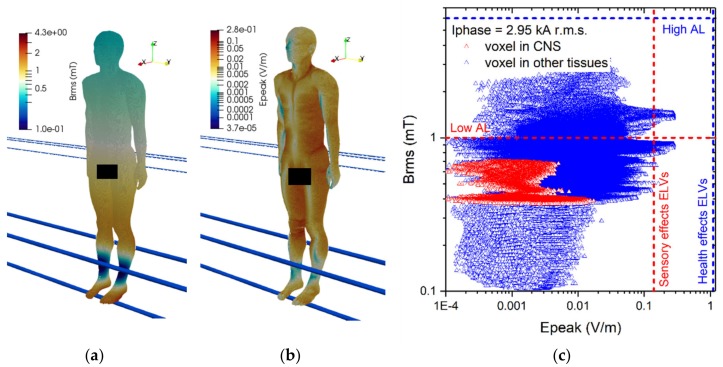
Results for posture A.1. On the left (**a**) the spatial distribution of the B-field amplitude (r.m.s. values in millitesla) over the body surface. In the middle (**b**) the corresponding spatial distribution of the E-field amplitude (peak values in volt per metre) over the body surface. On the right (**c**) the distribution of the voxel values.

**Figure 7:**
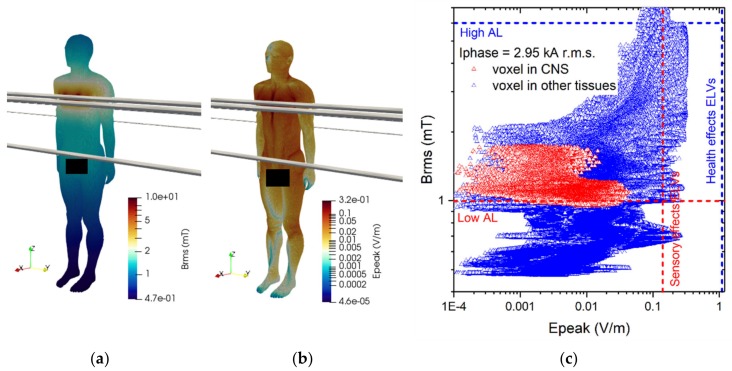
Results for posture A.2. On the left (**a**) the spatial distribution of the B-field amplitude (r.m.s. values in millitesla) over the body surface. In the middle (**b**) the corresponding spatial distribution of the E-field amplitude (peak values in volt per metre) over the body surface. On the right (**c**) the distribution of the voxel values.

**Figure 8:**
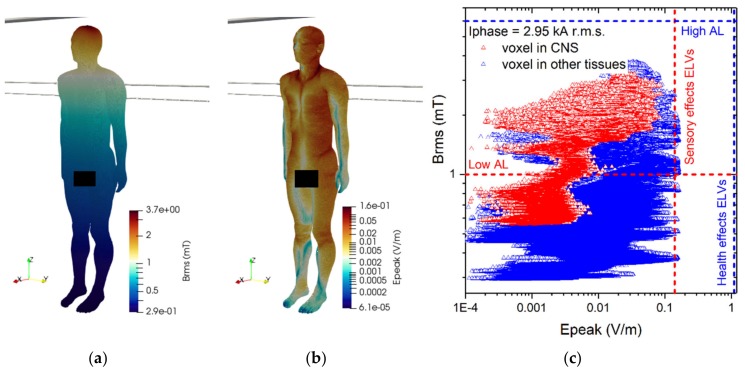
Results for posture A.3. On the left (**a**) the spatial distribution of the B-field amplitude (r.m.s. values in millitesla) over the body surface. In the middle (**b**) the corresponding spatial distribution of the E-field amplitude (peak values in volt per metre) over the body surface. On the right (**c**) the distribution of the voxel values.

**Figure 9:**
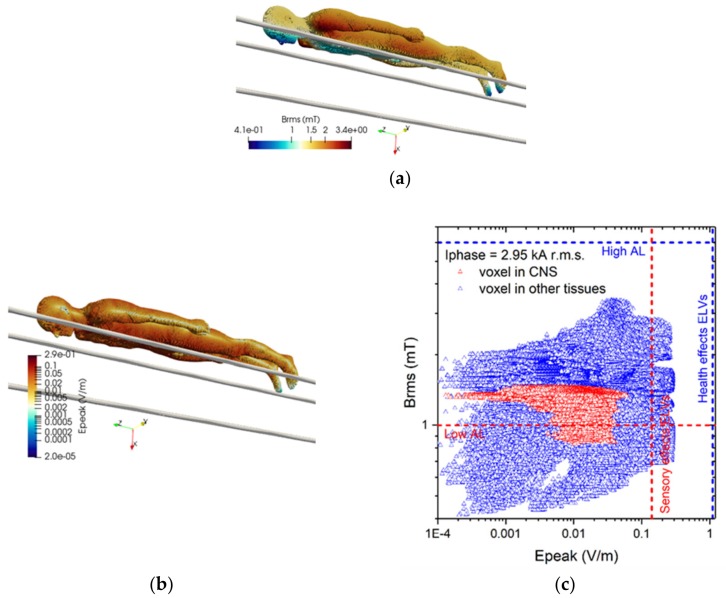
Results for posture A.4. On the upper left (**a**) the spatial distribution of the B-field amplitude (r.m.s. values in millitesla) over the body surface. On the lower left (**b**) the corresponding spatial distribution of the E-field amplitude (peak values in volt per metre) over the body surface. On the right (**c**) the distribution of the voxel values.

**Figure 10:**
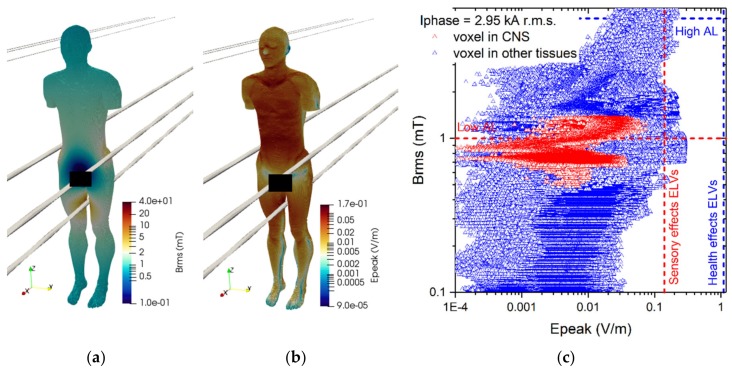
Results for posture A.5. On the left (**a**) the spatial distribution of the B-field amplitude (r.m.s. values in millitesla) over the body surface. In the middle (**b**) the corresponding spatial distribution of the E-field amplitude (peak values in volt per metre) over the body surface. On the right (**c**) the distribution of the voxel values.

The results of simulations related to postures A.1 to A.5 are collected in Figure 6, Figure 7, Figure 8, Figure 9 and Figure 10. These results refer to the ***A***-*ϕ* solver after outlier removal and with a safety factor of ~2 and are obtained assuming a phase current of 2.95 kA (r.m.s.). For each exposure condition, in the figures it is represented the spatial distribution of the B-field amplitude over the body surface and the corresponding surface values of the induced E-field amplitude. 

**Figure 11:**
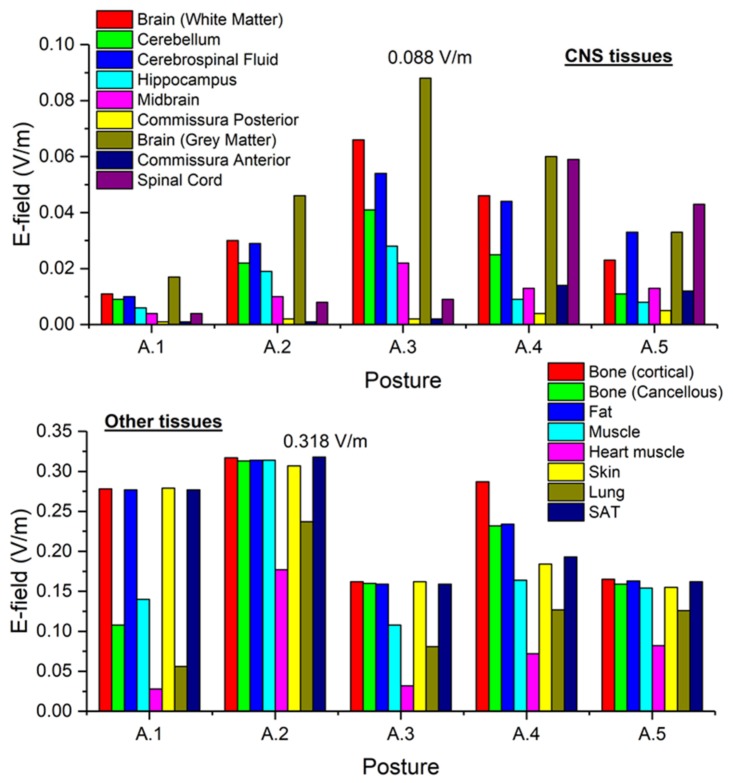
Exposure scenario A: on the upper diagram, maximum values of E-field (V/m) for the CNS tissues, on the bottom diagram, maximum values of E-field (V/m) for a selection of other tissues.

The maximum values of B-field and E-field are not spatially correlated, depending on the current paths that are determined by the interaction between the source field and the tissue properties within the body. Histograms in Figure 11 collect, for all postures of exposure scenario A, the maximum values of E-field respectively obtained in the CNS tissues and in all other tissues.

For posture A.1 the maximum B-field values are reached in the feet and in the knee regions, while the maximum E-field is found in the brain grey matter (0.017 V/m), for the CNS tissues, and in the bone cortical (0.278 V/m) and skin (0.279 V/m) for all other tissues. The B-field and E-field values associated to each body voxel are plotted in Figure 6c, separating the voxels belonging to CNS tissues and the voxels of the remaining tissues. The E-field peak values (resp. B-field r.m.s. values) in CNS ranges from about 10^−4^ V/m (resp. 0.38 mT) to 0.02 V/m (resp. 0.7 mT). The ELVs for CNS tissues and all other tissues are never exceeded.

For posture A.2 the maximum B-field values are reached in the thorax region, while the maximum E-field is found in the brain grey matter (0.046 V/m), for the CNS tissues, and in the bone cortical (0.317 V/m) and SAT (0.318 V/m) for all other tissues. The B-field and E-field values associated to each body voxel are plotted in Figure 7c, separating the voxels belonging to CNS tissues and the voxels of the remaining tissues. The E-field peak values (resp. B-field r.m.s. values) in CNS ranges from about 10^−4^ V/m (resp. 0.9 mT) to 0.05 V/m (resp. 1.8 mT). The ELVs for CNS tissues and all other tissues are never exceeded (despite the ALs are exceeded, in non-CNS tissues).

For posture A.3 the maximum B-field values are reached in the head region, while the maximum E-field is found in the brain grey matter (0.088 V/m), for the CNS tissues, and in the bone cortical (0.162 V/m) and skin (0.162 V/m) for all other tissues. The B-field and E-field values associated to each body voxel are plotted in Figure 8c, separating the voxels belonging to CNS tissues and the voxels of the remaining tissues. The E-field peak values (resp. B-field r.m.s. values) in CNS ranges from about 10^−4^ V/m (resp. 0.57 mT) to 0.1 V/m (resp. 3.8 mT). The ELVs for CNS tissues and all other tissues are never exceeded.

For posture A.4 the maximum B-field values are reached along the arms, while the maximum E-field is found in the brain grey matter (0.06 V/m), for the CNS tissues, and in the bone cortical (0.287 V/m) for all other tissues. The B-field and E-field values associated to each body voxel are plotted in Figure 9c, separating the voxels belonging to CNS tissues and the voxels of the remaining tissues. The E-field peak values (resp. B-field r.m.s. values) in CNS ranges from about 10^−4^ V/m (resp. 0.8 mT) to 0.06 V/m (resp. 1.7 mT). The ELVs for CNS tissues and all other tissues are never exceeded.

For posture A.5 the maximum B-field values are reached in the legs and in the side region, while the maximum E-field is found in the spinal cord (0.043 V/m), for the CNS tissues, and in the bone cortical (0.165 V/m) for all other tissues. The B-field and E-field values associated to each body voxel are plotted in Figure 10c, separating the voxels belonging to CNS tissues and the voxels of the remaining tissues. The E-field peak values (resp. B-field r.m.s. values) in CNS ranges from about 10^−4^ V/m (resp. 0.5 mT) to 0.07 V/m (resp. 1.5 mT). The ELVs for CNS tissues and all other tissues are never exceeded (despite the ALs for non-CNS tissues are exceeded).

### 3.3. Results about the Exposure Scenario B

**Figure 12:**
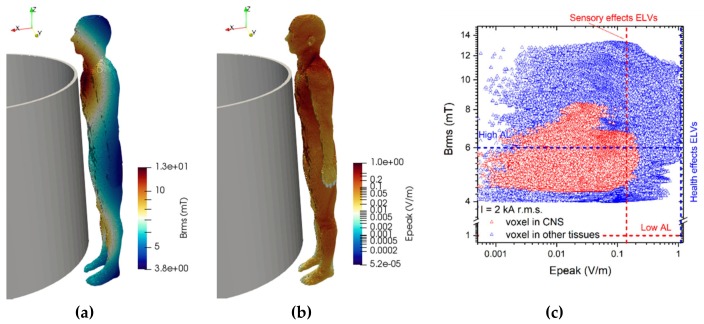
Results for posture B.1. On the left the (**a**) spatial distribution of the B-field amplitude (r.m.s. values, in millitesla) over the body surface. In the middle (**b**) the corresponding spatial distribution of the E-field amplitude (peak values, in volt per metre) over the body surface. On the right (**c**) the distribution of the voxel values.

**Figure 13:**
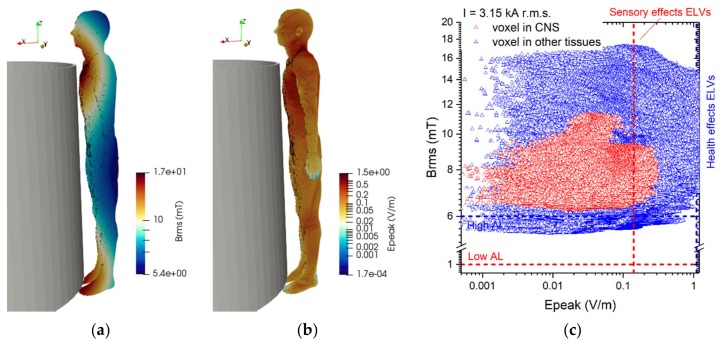
Results for posture B.2. On the left (**a**) the spatial distribution of the B-field amplitude (r.m.s. values, in millitesla) over the body surface. In the middle (**b**) the corresponding spatial distribution of the E-field amplitude (peak values, in volt per metre) over the body surface. On the right (**c**) the distribution of the voxel values.

The results of simulations related to postures B.1 and B.2 are shown in Figure 12 and Figure 13. 

**Figure 14:**
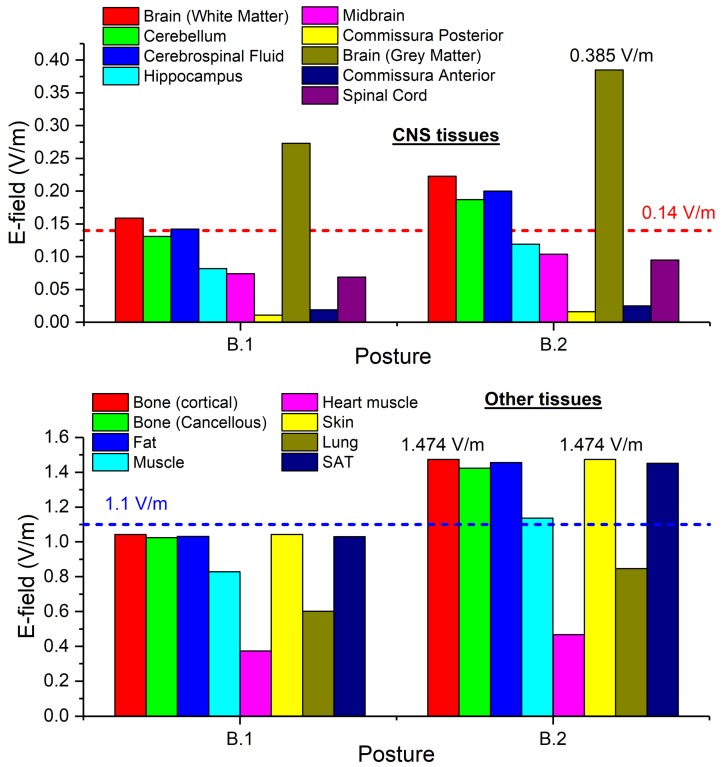
Exposure scenario B: on the upper diagram, maximum values of E-field (V/m) for the CNS tissues, on the bottom diagram, maximum values of E-field (V/m) for a selection of other tissues.

The reported results refer to the ***A***-*ϕ* solver after outlier removal and with a safety factor of ~2 and are obtained assuming a current of 2 kA (resp. 3.15 kA) (r.m.s.) for the 220 kV (resp. 380 kV) line trap coil. The figures show the spatial distribution of the B-field amplitude over the body surface and the corresponding surface values of the induced E-field amplitude. Histograms in Figure 14 collect, for both postures of exposure scenario B, the maximum values of E-field respectively obtained in the CNS tissues and all other tissues.

For both postures the maximum B-field values are reached in the thorax and neck regions, while the maximum E-field is found in the brain grey matter (0.159 V/m for B.1 and 0.223 V/m for B.2), for the CNS tissues, and in the bone cortical and in the skin (1.042 V/m for B.1 and 1.474 V/m for B.2) for all other tissues. The B-field and E-field values associated to each body voxel are plotted in Figure 12c and Figure 13c, separating the voxels belonging to CNS tissues and the voxels of the remaining tissues. For the 220 kV line trap coil the E-field peak values (resp. B-field r.m.s. values) in CNS ranges from 5×10^−4^ V/m (resp. 5.5 mT) to 0.3 V/m (resp. 8.5 mT). For the 380 kV line trap coil the E-field peak values (resp. B-field r.m.s. values) in CNS ranges from 5×10^−4^ V/m (resp. 7 mT) to 0.35 V/m (resp. 11.5 mT). The limits in CNS tissues are exceeded both for E and B-field for both postures. For case B.2, the limits are exceeded also in other tissues.

### 3.4. Results Concerning Exposure Scenario C

**Figure 15:**
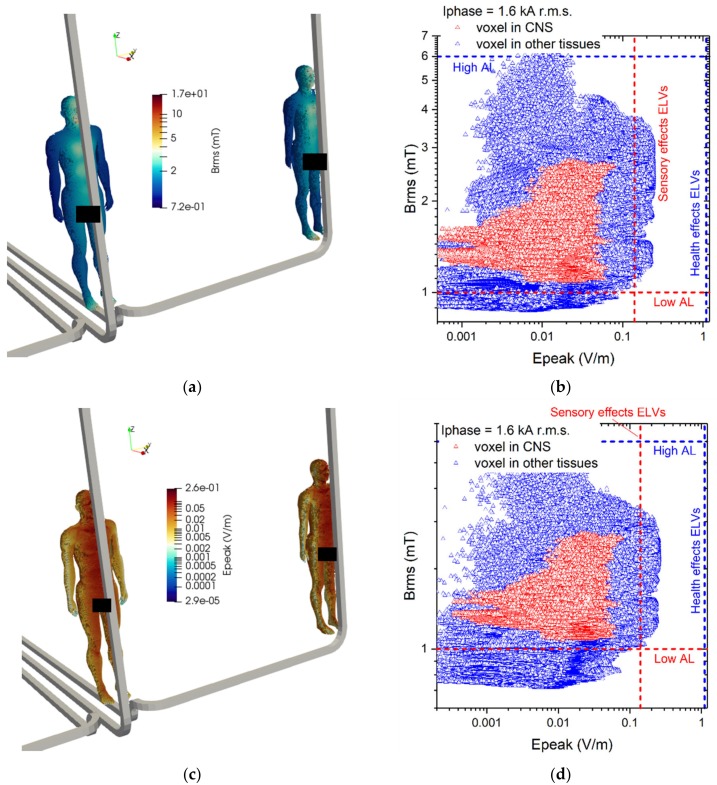
Results for posture C.1. On the upper left (**a**) the spatial distribution of the B-field amplitude (r.m.s. values, in millitesla) over the body surface for the two body positions. On the lower left (**b**) the corresponding spatial distribution of the E-field amplitude (peak values, in volt per metre) over the body surface. On the right the distributions of the voxel values: upper diagram (**c**) corresponding to the posture C.1a, lower diagram (**d**) corresponding to the posture C.1b.

**Figure 16:**
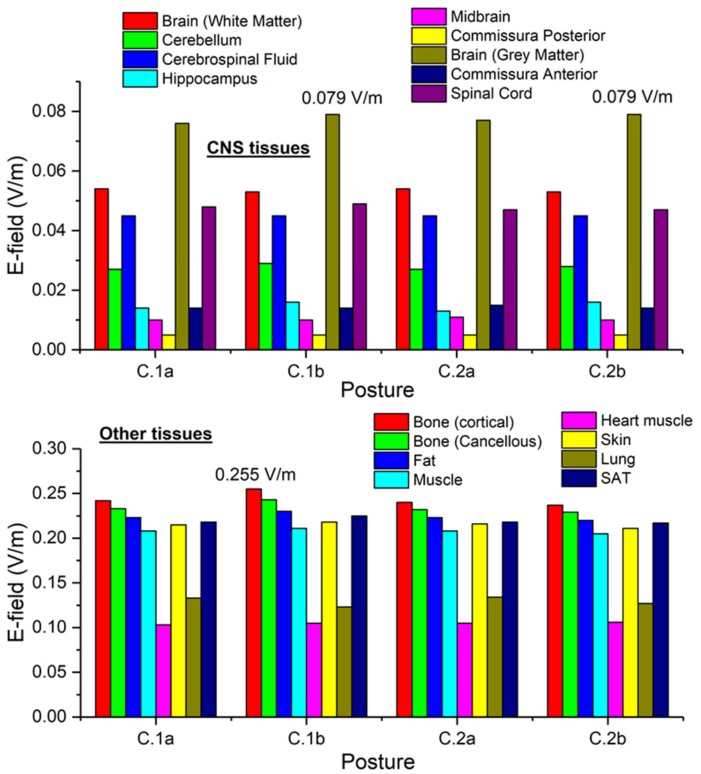
Exposure scenario C: on the upper diagram, maximum values of E-field (V/m) for the CNS tissues, on the bottom diagram, maximum values of E-field (V/m) for a selection of other tissues.

The results of simulations related to postures C.1 are collected in Figure 15. For each conductor configuration, results for both positions of the body are reported (close to the central and to the lateral conductor). The reported results refer to the ***A***-*ϕ* solver after outlier removal and with a safety factor of ~2 and are obtained assuming a phase current of 1.6 kA (r.m.s.). The figures show the spatial distribution of the B-field amplitude over the body surface and the corresponding surface values of the induced E-field amplitude. Histograms in Figure 16 collect, for all postures of exposure scenario C, the maximum values of E-field respectively obtained in the CNS tissues and all other tissues.

For all postures the maximum B-field values are reached in the thorax and head regions, while the maximum E-field is found in the brain grey matter (0.079 V/m), for the CNS tissues, and in the bone cortical (0.255 V/m) for all other tissues. The B-field and E-field values associated to each body voxel are plotted in Figure 15c,d, separating the voxels belonging to CNS tissues and the voxels of the remaining tissues. The E-field peak values (resp. B-field r.m.s. values) in CNS ranges from 5·10^−4^ V/m (resp. 1.1 mT) to 0.08 V/m (resp. 2.8 mT). The ELVs for CNS tissues and all other tissues are never exceeded. No appreciable differences are found for posture C.2, both in terms of spatial distribution and maximum values of the B-field and E-field values.

### 3.5. Analysis of Sensitivity to Body-Conductor Relative Position

The relative position between human body and conductors and, in particular, the minimum distance adopted in the previous analysis has been estimated on the basis of the standard operating conditions. A sensitivity to these parameters is analysed in this section in order to get the significance of the outcomes and an estimate of the applicability to a wider range of exposure conditions. 

In particular, the cases below reported have been analysed:

(a) For the exposure scenario A, posture A.3 that gives rise to the worst exposure conditions. The human body has been moved in order to reduce (resp. increase) the distance between the worker’s head and the closest conductor at 25 mm (resp. 75 mm). These cases are identified as A.3#1 and A.3#2, respectively. Moreover, the body has been moved along the transversal direction of conductors (*x*-axis in Figure 1) of−50 mm (case A.3#3) and + 50 mm (case A.3#4).
(b) For the exposure scenario B, the case of 380 kV line trap coil (B.2), that gives rise to the worst exposure conditions. The human body has been moved in order to reduce (resp. increase) the distance between the worker’s thorax and the coil at 5 mm (resp. 15 mm). These cases are identified as B.2#1 and B.2#2, respectively. Moreover, the body has been moved along the vertical axis (*z*-axis in Figure 2) of −200 mm (case B.2#3) and of +200 mm (case B.2#4).
(c) For the exposure scenario C, the case of phase conductors at a distance of 2.5 m and the worker placed in front of the lateral conductor (C.1b), that gives rise to the worst exposure conditions. The human body has been moved in order to reduce (resp. increase) the distance between the worker’s thorax and the coil at 30 mm (resp. 110 mm). These cases are identified as C.1b#1 and C.1b#2, respectively.

Considering that, for all analyzed scenarios, the limits related to sensory effects are the most severe (being reached within the human body before the health effect ELVs), the E-field values summarized in the following Table 4, Table 5 and Table 6 are limited to the CNS tissues, only.

For exposure A.3, the largest variations (from + 18% to −13%) of the maximum E-field values (found in the grey matter) are found varying the distance between the body and the conductors (cases A.3#1 and A.3#2). Moving the body along the transversal direction (*x*-axis) is less effective, giving rise to a variation of the maximum E-field of about −5%. In any case, the sensory ELVs are never exceeded.

For exposure B.2, the largest variation of the maximum E-field is found when moving the body along the vertical axis (cases B.2#3 and B.2#4). The value increases of 50% or reduces of 36%. The variation of the minimum distance between head and conductor from 5 mm to 15 mm does not appreciably affect the maximum E-field values (variations around 1%). The sensory ELVs still remain exceeded in the same CNS tissues.

For exposure C.1b, reducing the minimum distance between head and conductor from 70 mm to 30 mm causes a 40% increase of the maximum E-field value detected in the grey matter. Increasing the distance from 70 mm to 110 mm causes a 22% reduction of the maximum E-field value. It must be noted that for case C.1b#1 the sensory ELVs are not exceeded.

## 4. Conclusions

In this paper, the exposure of live-line workers to the magnetic fields has been analysed through a comprehensive dosimetric analysis using highly defined anatomical models and two alternative solvers for checking the consistency of the results. For each considered exposure scenario, the induced electric fields within the workers’ body have been compared with the sensory and health effects ELVs provided by the European Directive 2013/35/EU.

A set of relevant exposure scenarios has been considered, including exposure of workers near HV overhead lines and exposure of workers in substations (either near line trap coils or cable connections). These scenarios include positions where the HV operators work with bare hand method and they are nearly close to the source and positions where the operators make a check on HV cables.

The study on the three cases allows us to put forth the conclusions below:

- For scenario (A) the results obtained showed that the worst position is when the conductors are placed near the operator’s head, assuming a conductor - head distance equal to 50 mm. Considering a value of current equal to 2950 A r.m.s, in any analyzed position the ELVs for CNS tissues and all other tissues are never exceeded, despite in some cases the ALs are exceeded in non-CNS tissues. Considering this high current value, which is not normally achieved on overhead lines in Italy, it is not necessary to take further precautions for workers.
- For Scenario (B), only the typical work positions for the 220 kV and 380 kV voltage level have been studied. In both cases the parts of the body that are mainly involved are chest, neck and head. If the current flowing through the line trap exceeds the value of 1000 A, the E-field peak values could be higher than the regulatory limits. At present, in the Italian experience, this kind of job is actually done in conditions of out of service of the power line (dead working). In case of live-line working, the results obtained suggest the requirement that the activity is carried on with a continuous monitoring of the current values, in order to immediately stop the work if the value of 1000 A is exceeded.
- For scenario (C) the worst position, from the point of view of the exposure to the magnetic field, is the one with the operator in front of a lateral conductor. The E-field peak values induced in the body, with a current value equal to 1600 A r.m.s. used for the computation, are always found lower than regulatory limits. It is therefore not necessary to take further precautions for workers.

## Figures and Tables

**Table 1:** Electrical properties of some tissues.

Tissue	Electrical Conductivity (S/m)
**Central nervous system (CNS)**	
Brain (White Matter)	0.265
Cerebellum	0.660
Cerebrospinal Fluid	1.777
Hippocampus	0.276
Midbrain	0.234
Commissura Posterior	0.265
Brain (Grey Matter)	0.239
Commissura Anterior	0.265
Spinal Cord	0.234
**Other tissues**	
Bone (Cortical)	0.0035
Bone (Cancellous)	0.0821
Fat	0.0573
Muscle	0.355
Heart Muscle	0.381
Skin	0.170
Lung	0.105
Subcutaneous Adipose Tissue (SAT)	0.057

**Table 2:** Parameters of the considered line trap coils.

Voltage (kV)	Diameter (mm)	Height (mm)	Number of Turns
220	1090	1120	17
380	1450	1350	15

**Table 3:** Maximum values of E-field obtained for the two solvers after filtering for outlier removal (E_out_T_ and E_out_A-_*_ϕ_*). The values filtered with the 99th percentile are also reported for both solvers (E_99_T_ and E_99_A-_*_ϕ_*).

Posture	E-Field After Outlier Removal			E-Field 99th Percentile		
	E_out_A-_*_ϕ_* (V/m)	E_out_T_ (V/m)	Ratio E_out_T_/E_out_A-_*_ϕ_*	E_99_A-_*_ϕ_* (V/m)	E_99_T_ (V/m)	Ratio E_99_T_/E_99_A-_*_ϕ_*
A.1	0.279	0.201	**0.72**	0.060	0.057	0.95
A.2	0.318	0.354	1.11	0.096	0.103	1.07
A.3	0.162	0.259	1.60	0.060	0.065	**1.08**
A.4	0.288	0.285	0.99	0.076	0.077	1.01
A.5	0.166	0.221	1.34	0.071	0.066	**0.93**
B.1	1.042	1.616	1.55	0.414	0.441	1.07
B.2	1.474	2.192	1.49	0.578	0.611	1.06
C.1a	0.243	0.393	**1.62**	0.082	0.083	1.01
C.1b	0.255	0.364	1.42	0.081	0.083	1.03
C.2a	0.240	0.370	1.54	0.083	0.083	1.00
C.2b	0.238	0.347	1.46	0.082	0.083	1.01

Note: The maximum variation between E_out_T_ and E_out_A-_*_ϕ_* are evidenced in bold character.

**Table 4:** Maximum values of E-field (V/m) for the CNS tissues. Influence of body position for posture A.3.

Tissue	Reference Position (A.3)	Case A.3#1	Case A.3#2	Case A.3#3	Case A.3#4
Brain (White Matter)	0.066	0.080	0.057	0.055	0.062
Cerebellum	0.041	0.045	0.037	0.038	0.041
Cerebrospinal Fluid	0.054	0.067	0.048	0.052	0.054
Hippocampus	0.028	0.031	0.026	0.025	0.030
Midbrain	0.022	0.025	0.020	0.022	0.019
Commissura Posterior	0.002	0.002	0.002	0.002	0.002
Brain (Grey Matter)	**0.088**	**0.104**	**0.077**	**0.085**	**0.084**
Commissura Anterior	0.002	0.002	0.002	0.003	0.001
Spinal Cord	0.009	0.009	0.008	0.009	0.009

Note: The variation of the maximum E-field values reached in the Grey Matter is highlighted in bold character.

**Table 5:** Maximum values of E-field (V/m) for the CNS tissues. Influence of body position for posture B.2. The variation of the maximum E-field values reached in the Grey Matter is highlighted in bold character.

Tissue	Reference Position (B.2)	Case B.2#1	Case B.2#2	Case B.2#3	Case B.2#4
Brain (White Matter)	0.223	0.220	0.226	0.334	0.155
Cerebellum	0.187	0.184	0.189	0.247	0.126
Cerebrospinal Fluid	0.200	0.197	0.202	0.291	0.150
Hippocampus	0.119	0.118	0.120	0.184	0.075
Midbrain	0.104	0.102	0.105	0.126	0.073
Commissura Posterior	0.016	0.016	0.016	0.017	0.010
Brain (Grey Matter)	**0.385**	**0.382**	**0.389**	**0.576**	**0.245**
Commissura Anterior	0.025	0.025	0.026	0.015	0.019
Spinal Cord	0.095	0.093	0.097	0.063	0.080

Note: The variation of the maximum E-field values reached in the Grey Matter is highlighted in bold character.

**Table 6:** Maximum values of E-field (V/m) for the CNS tissues. Influence of body position for posture C.1b.

Tissue	Reference Position (C.1b)	Case C.1b#1	Case C.1b#2
Brain (White Matter)	0.053	0.067	0.044
Cerebellum	0.029	0.035	0.024
Cerebrospinal Fluid	0.045	0.064	0.037
Hippocampus	0.016	0.021	0.014
Midbrain	0.010	0.011	0.009
Commissura Posterior	0.005	0.005	0.004
Brain (Grey Matter)	**0.079**	**0.112**	**0.062**
Commissura Anterior	0.014	0.017	0.012
Spinal Cord	0.049	0.057	0.042

Note: The variation of the maximum E-field values reached in the Grey Matter is highlighted in bold character.
